# Open and reproducible research in musculoskeletal imaging: why it matters and how to implement it with the guidelines of the Open and Reproducible Musculoskeletal Imaging Research (ORMIR) community

**DOI:** 10.1093/jbmrpl/ziag025

**Published:** 2026-02-20

**Authors:** Serena Bonaretti, Mojtaba Barzegari, Melissa Bevers, Steven Boyd, Andrew J Burghardt, Donnie Cameron, Francesco Chiumento, Gianluigi Crimi, Gerald Degenhart, Pholpat Durongbhan, Michelle Alejandra Espinosa Hernandez, Giulia Fraterrigo, Ali Ghasem-Zadeh, Lorenzo Grassi, Jukka Hirvasniemi, Seyedmahdi Hosseinitabatabaei, Gianluca Iori, Joeri Kok, Michael Kuczynski, YoungJun Lee, Cecilia Liberati, Sarah Manske, Matt McCormick, Maria Monzon, Martino Pani, Simone Poncioni, Jilmen Quintiens, Sabine Räuber, Paul Ritsche, Alfonso Dario Santamaria, Francesco Santini, Fabio Sarto, Enrico Schileo, Vincent Stadelmann, Kathryn S Stok, Rachel Surowiec, Fulvia Taddei, Jared Vicory, Matthias Walle, Mariska Wesseling, Danielle Whittier, Bettina Willie, Andy Kin On Wong, Dženan Zukić

**Affiliations:** Independent Researcher, Zurich, 8000, Switzerland; Department of Chemical Engineering and Chemistry, Eindhoven University of Technology (TU/e), Eindhoven, 5612 AZ, The Netherlands; Department of Internal Medicine, VieCuri Medical Center, Venlo, 5912 BL, The Netherlands; NUTRIM Institute of Nutrition and Translational Research In Metabolism, Maastricht University, Maastricht, 6229 ER, The Netherlands; Department of Biomedical Engineering, Eindhoven University of Technology, Eindhoven, 5612 AZ, The Netherlands; Department of Radiology, Cumming School of Medicine, University of Calgary, Calgary, AB T2N 2T8, Canada; McCaig Institute for Bone and Joint Health, University of Calgary, Calgary, AB T2N 4Z6, Canada; Department of Radiology & Biomedical Imaging, University of California, San Francisco, San Francisco, CA 94143, United States; Department of Medical Imaging, Radboud University Medical Center, Nijmegen, 6525 GA, The Netherlands; School of Electronic Engineering, Dublin City University (DCU), Dublin, D09 V209, Ireland; Bioengineering and Computing Laboratory, IRCCS Istituto Ortopedico Rizzoli, Bologna, 40136, Italy; Core Facility MicroCT, University Clinic for Radiology, Medical University Innsbruck, Innsbruck, 6020, Austria; Department of Biomedical Engineering, The University of Melbourne, Carlton, VIC 3053, Australia; Department of Biomedical Engineering, The University of Melbourne, Carlton, VIC 3053, Australia; Rehabilitation Sciences Institute, University of Toronto, Toronto, ON M5G 1V7, Canada; Bioengineering and Computing Laboratory, IRCCS Istituto Ortopedico Rizzoli, Bologna, 40136, Italy; Departments of Endocrinology and Medicine, Austin Health, The University of Melbourne, Heidelberg, VIC 3084, Australia; Department of Biomedical Engineering, Lund University, Lund, 223 63, Sweden; Department of Radiology & Nuclear Medicine, Erasmus MC University Medical Center Rotterdam, Rotterdam, 3015 GD, The Netherlands; Department of Bioengineering, Faculty of Engineering, McGill University, Montreal, QC H3A 0E9, Canada; Center for Photon Science, Paul Scherrer Institute PSI, Villigen, 5232, Switzerland; Department of Biomedical Engineering, Eindhoven University of Technology, Eindhoven, 5612 AZ, The Netherlands; Department of Cell Biology and Anatomy, Cumming School of Medicine, University of Calgary, Calgary, AB T2N 2T8, Canada; McCaig Institute for Bone and Joint Health, University of Calgary, Calgary, AB T2N 4Z6, Canada; Weldon School of Biomedical Engineering, Purdue University, West Lafayette, IN 47907, United States; Department of Mechanical Engineering, KU Leuven, Leuven, 3001, Belgium; Department of Radiology, Cumming School of Medicine, University of Calgary, Calgary, AB T2N 2T8, Canada; McCaig Institute for Bone and Joint Health, University of Calgary, Calgary, AB T2N 4Z6, Canada; Fideus Labs, Durham, NC 27713, United States; Department of Health Science and technology, ETH Zurich, Zurich, 8092, Switzerland; School of Electrical and Mechanical Engineering, University of Portsmouth, Portsmouth, PO1 3DJ, United Kingdom; ARTORG Center for Biomedical Engineering Research, University of Bern, Bern, 3008, Switzerland; Department of Mechanical Engineering, KU Leuven, Leuven, 3001, Belgium; Department of Biomedical Engineering, University of Basel, Basel, 4123, Switzerland; Department of Sport, Exercise and Health, University of Basel, Basel, 4052, Switzerland; Bioengineering and Computing Laboratory, IRCCS Istituto Ortopedico Rizzoli, Bologna, 40136, Italy; Department of Biomedical Engineering, University of Basel, Basel, 4123, Switzerland; Department of Biomedical Sciences, University of Padova, Padova, 35131, Italy; Bioengineering and Computing Laboratory, IRCCS Istituto Ortopedico Rizzoli, Bologna, 40136, Italy; Department of Research and Development, Schulthess Klinik, Zurich, 8008, Switzerland; Department of Biomedical Engineering, The University of Melbourne, Carlton, VIC 3053, Australia; Weldon School of Biomedical Engineering, Purdue University, West Lafayette, IN 47907, United States; Bioengineering and Computing Laboratory, IRCCS Istituto Ortopedico Rizzoli, Bologna, 40136, Italy; Medical Computing, Kitware, Inc., Carrboro, NC 27510, United States; McCaig Institute for Bone and Joint Health, University of Calgary, Calgary, AB T2N 4Z6, Canada; Department of Orthopedics and Sports Medicine, Erasmus Medical Center, Rotterdam 3000 CA, The Netherlands; McCaig Institute for Bone and Joint Health, University of Calgary, Calgary, AB T2N 4Z6, Canada; Department of Cell Biology and Anatomy, Cumming School of Medicine, University of Calgary, Calgary, AB T2N 2T8, Canada; Faculty of Dental Medicine and Oral Health Sciences, McGill University and Shriners Hospital for Children-Canada, Montreal, QC H4A 0A9, Canada; Joint Department of Medical Imaging; Schroeder Arthritis Institute, University Health Network, Toronto, ON M5G 2C4, Canada; Dalla Lana School of Public Health, University of Toronto, Toronto, ON M5T 3M7, Canada; Medical Computing, Kitware, Inc., Carrboro, NC 27510, United States

**Keywords:** Open research, reproducible research, data sharing, open-source code, computational narratives, guidelines, license, ORMIR

## Abstract

The Open and Reproducible Musculoskeletal Imaging Research community is a scientific community dedicated to promoting openness and reproducibility in musculoskeletal imaging, image processing, and computational modeling. In this perspective paper, we outline the motivations for conducting transparent research and provide practical guidelines for implementing it. We start by defining open and reproducible research and describing the benefits and challenges of working transparently. Next, we redefine the outputs of a computational research study as—ideally—a combination of data, code, and a publication, recommend a folder and file structure that reflects these three study outcomes, and describe how to maintain and update such a structure during the study and at study publication. Finally, we emphasize that working in an open and reproducible manner is a learning process, and the best way to acquire the necessary competencies is simply to start.

## Graphical Abstract

**Graphical Abstract:**
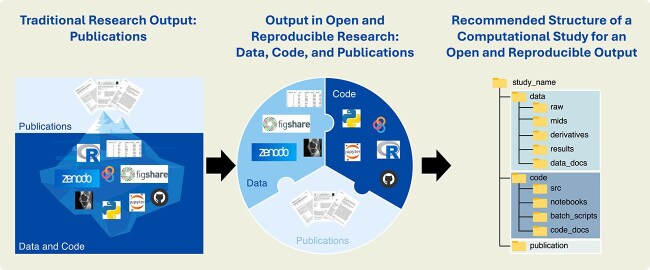

## Introduction

In 2012, Panagiotopoulou *et al.*^1^ published an article titled “What makes an accurate and reliable subject-specific finite element model? A case study of an elephant femur.” In this work, they sought “to construct the first reliable finite element model of the femur of an adult Asian elephant” from CT images. As part of the study, the authors conducted convergence analyses for the size, type, and number of elements used in the geometry discretization of the femur anatomy. In the following years, the paper received 33 citations (retrieved from Google Scholar: https://scholar.google.com. Accessed July 21, 2025). Some of these publications referred to the methodology and findings of this study in their introduction or discussion, while other publications built upon the study methodology and findings. Among the latter, in 2018, Brassey *et al.*^2^ published a study in which they used the same size, type, and number of elements as in Panagiotopoulou’s publication, motivating that “previous research *has found* such meshes perform well” and “strain values *have been found* to converge when element numbers exceed 200,000.” While building upon previous work is typical in research, in this case, there was an unexpected outcome. In 2014—four years before Brassey’s publication—Panagiotopoulou’s paper had been self-retracted because the authors “uncovered problems with the methods [...] and, therefore, some of the final data”.^3^

This story highlights three important considerations. First, Panagiotopoulou *et al.* deserve high praise for their scientific integrity. Second, what occurred to Brassey *et al.* could happen to any researcher who builds a study on previous findings. Third, we researchers can be inspired to ask fundamental questions about some practical needs that we face in our daily work: How can we and our collaborators create and publish verifiable methodologies and findings that others can build upon? How can we avoid building our studies on incorrect methods and results? And how can we check the correctness of methodologies and results when we review a paper and assess its scientific value? The answer to these questions is: open and reproducible research.

## Open and reproducible research: definitions, benefits, and challenges

Openness refers to “an approach to research based on open cooperative work that emphasizes the sharing of knowledge, results and tools as early and widely as possible”.^4^ Reproducibility—intended here as *computational* reproducibility—is the ability of researchers to duplicate the results of a previous study using the “same input data, computational steps, methods, and conditions of analysis”.^5^ Together, openness and reproducibility constitute a *way of working* whose ultimate goal is to improve the reliability and efficiency of scientific progress.^6^^,^^7^

The scientific advantages of open and reproducible research are several and include evaluating the correctness of scientific claims,^8^ building on previous work with confidence and efficiency^9^—by reusing and extending existing computational pipelines—and comparing and validating new methods and algorithms against existing ones without having to reimplement them.^10^ These scientific benefits are accompanied by academic advantages. Publishing open data can increase the number of citations by approximately 70%, regardless of journal impact factor, author country of origin, and date of publication.^11^ Similarly, making code available online can increase citations by a factor of three.^12^ Furthermore, research laboratories can greatly benefit from adopting open and reproducible practices. Such a transparent methodological approach fosters an intrinsic “discipline” that supports the continuity of maintenance and extension of computational tools created in the labs, which would otherwise risk a short lifespan as they are usually developed by successive and distinct generations of PhD students and postdocs.^6^

Despite the aforementioned advantages, working in an open and reproducible manner has been challenging. Until a few years ago, guidelines were scarce, computational tools were not fully developed, and permanent, large, and free repositories for data and code were unavailable.^13^ However, nowadays there are numerous sources of information on open and reproducible research. The “Ten simple rules” series includes guidelines on good research practice,^14^ open development of scientific software,^15^ and effective research data management.^16^ Mature executable environments are available, such as Binder (https://mybinder.org), and established repositories, such as Zenodo (https://zenodo.org), facilitate the sharing of data and code. In addition to these practical challenges, many scientists were concerned about not receiving proper recognition, having to allocate additional time and effort to prepare data and code for release, and providing advantages to competing datasets or software when releasing seminal work.^17^ However, nowadays, there are funding programs for open science, supported by both agencies (eg, Canadian Institutes of Health Research) and foundations (eg, Chan Zuckerberg Initiative), and reward practices involving hiring criteria and credit of scholarly outputs.^18^ Despite these considerable advances, the most critical limitation to sharing data and code may be related to the way we conceive the outcomes of computational studies.

## What is the outcome of a computational research study?

**Figure 1:**
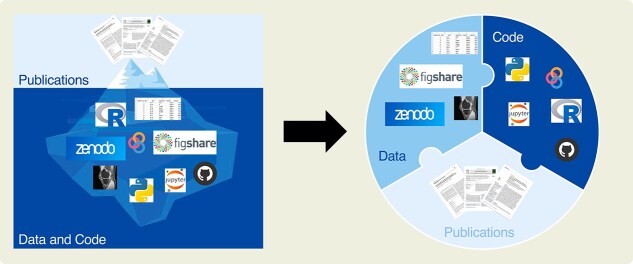
Representation of research outcomes. Traditionally, publications are the main research outcome. They constitute the shared, visible tip of an iceberg, whereas data and code remain hidden in the lab (left; modified from https://stodden.net/AMP2011/slides/2011-amp-reproducible-research.pptx.pdf). Conversely, in open and reproducible research data, code, and publication constitute the three interlocking pieces of the puzzle and are essential to each other to represent the entire study (right).

Traditionally, the main outcome of a computational research study is a scientific paper. As we know, papers are concise reports limited by a defined number of words (typically 3000-6000) and a restricted number of figures and tables (usually 3-6). To meet these constraints, only a selection of the methods and results developed and obtained during a study are presented in a paper. Crucial implementation details (eg, computational initializations and parameters) are often omitted,^12^ as well as intermediate or individual findings, because results are usually presented as aggregated statistics, such as means and standard deviations. In this context, a scientific paper does not represent “the scholarship itself, it’s merely scholarship advertisement”, as Donoho famously wrote in 2009.^6^ In other words, a scientific paper is just the tip of the iceberg representing research efforts.^19^ The actual work—that is, the data and code—remains hidden underwater (Figure 1, left).

In open and reproducible research, data and code emerge to be an integrative part of the outcome of a computational study. Together with publications, they constitute the three essential, interlocked pieces of the puzzle necessary to understand and reproduce the entire study (Figure 1, right). From the raw data of the input, through the code, researchers can recreate the results presented in the publication, and subsequently reuse and extend the workflow for new studies. However, for this to be feasible, data and code should be clearly structured and documented.^20^

More recently, numerous guidelines have become available on how to publish computational studies in an open and reproducible manner while enabling attribution and facilitating sharing.^8^^,^^9^^,^^15^^,^^21^^,^^22^ Moreover, researchers in many fields have formed scientific communities to establish consensus-based standards and develop computational tools specific to their disciplines.^23^^–^^25^

## ORMIR guidelines for open and reproducible computational studies in MSK imaging research

In musculoskeletal (MSK) imaging, the Open and Reproducible Musculoskeletal Imaging Research (ORMIR) community was founded in 2020, with the aims of creating open, reproducible, well-tested, and well-documented code to analyze MSK images; standardizing data acquisition and management to favor data sharing and algorithm comparison; and promoting a culture of openness and reproducibility in musculoskeletal imaging, image processing, and computational modelling for a faster advancement of the field.^26^ The community is currently composed of approximately 70 international researchers from academia and industry at various career stages who have been active in creating guidelines, templates, and software to manage and analyze MSK images. At the moment, the following working groups are active: biomechanics, high-resolution CT, knee, muscle, data management and sharing, and spine—see www.ormir.org.

**Figure 2:**
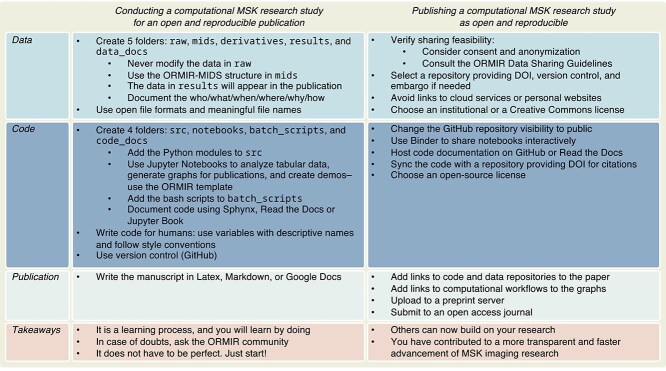
Summary of best practices for open and reproducible computational studies in musculoskeletal imaging research.

In the next two sections of this article, we will present the ORMIR guidelines on how to create an open and reproducible computational study using MSK images. These guidelines are based on the principle that open and reproducible research constitutes a methodological approach that should be implemented throughout an entire study, rather than being considered as a time- and resource-intensive task at publication. In addition, these guidelines are intended as a recommendation for future projects rather than strict rules, and they can be adapted to the specific needs of any computational study. ORMIR remains open to different folder structures that predate these guidelines or that are determined by project-specific requirements. In the following, first, we will focus on how to conduct a computational MSK research study for an open and reproducible publication. Then, we will provide suggestions on how to integrate and share the three study outcomes at publication. All recommendations are summarized in Figure 2.

## Conducting a computational MSK research study for an open and reproducible publication

**Figure 3:**
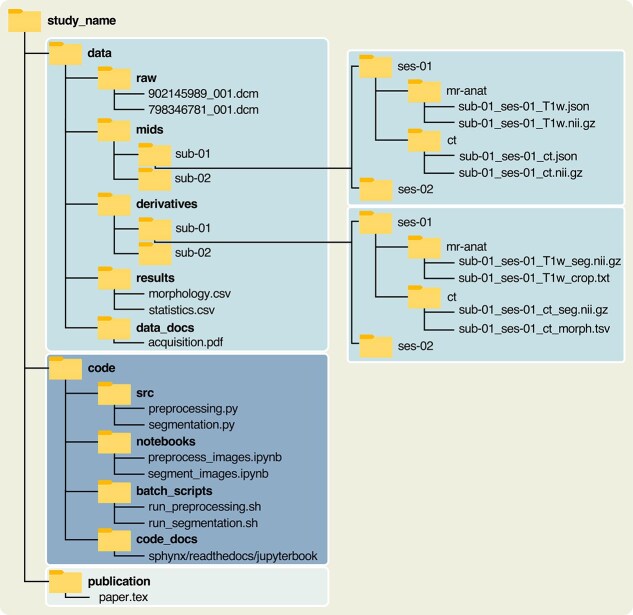
Folder and file structure of a computational research study for an open and reproducible publication. File extensions with acronyms expansions: .dcm: Digital Imaging and Communications in Medicine (standard file format for medical imaging data and associated metadata); .json: JavaScript Object Notation; .nii.gz: Neuroimaging Informatics Technology Initiative (compressed file format used to store medical imaging volumes); .txt: Plain Text; .tsv: Tab-Separated Values; .csv: Comma-Separated Values; .pdf: Portable Document Format; .py: Python Script; .ipynb: Interactive Python Notebook; .sh: Shell Script (script file containing commands for a Unix/Linux shell); and .tex: TeX (source file for documents written using the LaTeX typesetting system).

Begin by organizing your research project into a folder structure similar to the one in Figure 3. You can download the folder structure from Zenodo (www.doi.org/10.5281/zenodo.17206440) or implement it manually. Start with a main folder and name it after the study. Within the main folder, create three additional folders, each for one of the three study outcomes: data, code, and publication. The structure and content of each folder are described in more detail in the following sections.

### Data

Within the data folder, add five folders: raw, mids, derivatives, and results for data at different stages of the reproducible workflow, and data_docs for documentation.

In the folder raw, store the original data of the study, that is, the image files—usually in DICOM or proprietary format—and demographic or clinical information of the subjects—commonly in tabular format. These data constitute the input of your workflow. Do not modify them either via computational or manual intervention to ensure the reproducibility of the whole study.^8^^,^^27^

In the folder mids, restructure the files in raw following the specifications defined by the ORMIR Musculoskeletal Imaging Data Structure (ORMIR-MIDS),^28^ which extends and adapts the Brain Imaging Data Structure (BIDS) principles^29^ specifically for MSK imaging. The new structure comprises a hierarchy of nested folders arranged by subject, session, and imaging modality. It can be created using the ORMIR-MIDS Python package, which also converts the images from the Digital Imaging and Communications in Medicine (DICOM) format^30^ to the Neuroimaging Informatics Technology Initiative (NIfTI) format^31^ (Figure 3, right). The NIfTI format was chosen for compatibility with BIDS specifications and because it stores 3D/4D MSK imaging data into a single, standardized file, enabling efficient and reproducible processing.

During the study, in the folder derivatives, store the intermediate outputs of the computational workflow—such as segmentation masks, morphological measurements, and finite element analyses. Store also files that contain parameters used to modify the data contained in mids into the data contained in derivatives—eg, a .csv or .txt file containing the six parameters of a 3D image cropping. Saving these intermediate outputs will facilitate debugging and the potential reuse of partial findings.^9^

In the folder results, save the final output of the computational workflow, including tabular files reporting morphological, densitometric, mechanical, or statistical calculations across subjects. These are the data that you will present in the *Results* section of the publication.

Finally, in the folder data_docs, add the data documentation, such as acquisition protocol, ethical approval, and any metadata—that is, information about the data. As currently ORMIR does not provide specific guidelines for data documentation, simply collect all the reasonable information that specify the “who/what/when/where/why/how” of your dataset,^13^ so that other researchers can understand and reuse it. In general, provide information to make your data as FAIR (findable, accessible, interoperable, and reusable) as possible to maximize transparency, facilitate reuse, and support reproducibility.^32^

At every stage of the computational workflow, use open and widely supported file formats.^27^ For imaging data, prefer NIfTI or other open standards to scanner-specific proprietary formats. For tabular data, use comma-separated values (.csv) or tab-separated values (.tsv) over proprietary formats, such as Excel. Finally, give files meaningful names that clearly indicate their content.^27^

### Code

In the folder code, create the four folders src, notebooks, batch_scripts, and code_docs. In larger projects, add the additional folder tests for automated unit tests.^33^

In the folder src (abbreviation for source), add the core algorithms of the study. Write code in the open-source programming language Python, which has gained popularity in medical imaging for the availability of general-purpose image processing packages—such as ITK^34^ and SimpleITK^35^—as well as deep learning and machine learning packages—including PyTorch,^36^ TensorFlow,^37^ and Scikit-learn.^38^ Within src, organize your code into Python modules, that is, .py files. For larger projects, group the modules into a Python package and change the folder name to the package name, as required by the packaging tools.^39^ If you are more familiar with other programming languages, including open-source languages such as C++ or R, you may use them as well, provided that the code is well documented.

In the folder notebooks, add the study’s Jupyter Notebooks, which are the ideal format for integrating code with narrative, equations, and visualizations to effectively document reproducible workflows.^9^ Download the Jupyter Notebook template created by the ORMIR community from Zenodo (www.doi.org/10.5281/zenodo.17206440) and adapt it to your study. The template has a clear structure composed of aims, computational steps, outputs, and dependencies—that is, a print of the hardware characteristics and Python package versions.^9^ Use notebooks to analyze tabular data, generate figures for the publication, collect the main function calls from the modules in src into a computational workflow with narrative, and create examples or demos for your project.

In the folder batch_scripts, save the bash files. These files are the scripts that call the algorithms from the folder src and that you use for large-scale computations on servers.

In all cases, write code “for humans'', that is, make it readable rather than unnecessarily complex.^40^ To create clear code, use variables with descriptive names that reflect their role in the computation^40^ and adhere to the Python coding convention PEP8.^41^ Moreover, modularize code into functions—or classes—that represent units of operations.^9^^,^^27^ Document each function following the NumPy Style^42^ or Google Python Style^43^—which clearly describe functionality, inputs, and outputs—and add inline comments to explain non-obvious steps and clarify the underlying logic. Finally, synchronize the code to a repository like GitHub (https://github.com), GitLab (https://gitlab.com), or similar. Such repositories provide version control to preserve the history of changes and revert to previous code in case of errors, and they are convenient for coordinating programming tasks in collaborative software development.^21^

Finally, in the folder code_docs (or simply docs) create code documentation for developers and users, especially if you are building a large project.^44^ In the documentation for developers, carefully explain the technical details of the code. Create this documentation using Sphynx^45^ or Read the Docs^46^ as they are compatible with the NumPy and Google Python styles. In the documentation for users, explain in nontechnical language how to install and execute your code and provide examples or demos. Write this documentation using Jupyter Book.^47^

### Publication

In the last folder publication, store all the material relative to the scientific paper. Write the manuscript in a format that permits version control, such as LaTeX or Markdown, and opt for online tools that allow tracking changes and collaborative editing, such as Overleaf (www.overleaf.com), Google Docs (https://docs.google.com), Microsoft Word for the web (https://word.cloud.microsoft), or similar.^27^

## Publishing a computational MSK research study as open and reproducible

At publication, the primary task is to decide how and where to share the three study outcomes. Here are the details for each of them.

### Data

Sharing study data requires attention to two main aspects: assessing the feasibility of sharing and choosing a data repository.^48^^,^^49^

The feasibility of sharing depends on legal and ethical factors, including whether the study participants signed an appropriate consent form, the level of data anonymization or deidentification required, and the privacy legislation of your institution and country. These factors can preclude sharing some or all the content of the folders raw, mids, derivatives, and data_docs. However, you should be able to share the final aggregated data of your study—that is, the content of the folder results—which should not contain any personal data of the individual study participants. Further details are provided in the ORMIR Data Sharing Guidelines^50^ (www.ormir.org/data_sharing_guidelines), which aim to clarify the main aspects involved in sharing MSK imaging data. These guidelines include information about informed consent, data ownership, and other legal and ethical considerations. For example, the consent form page provides templates of generic consent forms, including examples that are compliant and non-compliant with the European General Data Protection Regulation (GDPR), as well as institutional consent forms from the University of Calgary, Hamilton Health Sciences in Ontario, the UK Biobank, and the National Institutes of Health (NIH). The guidelines also address the issue of data ownership, which remains an unresolved problem. In some cases, data belong to the researchers and/or academic institutions supporting the research. In other cases, data belong to funding agencies sponsoring the projects (eg, the NIH in the United States). In still other cases, data are considered to belong to the study participants, who may have the right to request the erasure of their personal paper and electronic research records, as stipulated by the European GDPR. In general, before sharing data, discuss this possibility with your Principal Investigator, your institution, or your local Ethical Committee, who can provide appropriate legal and ethical advice.

To date, only a few dedicated MSK repositories exist where you can share your data, such as the Universal Musculoskeletal Ultrasound Repository (UMUD).^51^ If your data does not fit the scope of such repositories, share the data in your institution repository, following their regulation. Otherwise, use general purpose repositories such as Zenodo (https://zenodo.org)—where ORMIR is present as a community label—or Figshare (https://figshare.com). Both these repositories provide a digital object identifier (DOI) to ensure permanent availability and enable citation, versioning for data, and an embargo period if you cannot immediately share your data. Avoid links to personal websites or cloud storage, because these links tend to expire or be deleted.^52^^,^^53^

### Code

To make your code open, simply change the repository visibility from private to public on GitHub (or a similar platform). Complete the README.md file of the repository using the template provided by the ORMIR community (www.doi.org/10.5281/zenodo.17206440). Whenever possible, share the notebooks in an interactive and reproducible cloud environment, such as Binder (https://mybinder.org).^54^ Host the code documentation created with Sphynx or Jupyter Book on GitHub Pages^55^^,^^56^ or the hosting service provided by Read the Docs. Finally, synchronize the GitHub repository with Zenodo,^57^ Figshare,^58^ or Software Heritage^59^ to obtain a DOI that enables reproducibility and citations. Note that sharing data and code alone may not be sufficient to guarantee inter-group computational reproducibility as software dependencies and execution environments may vary across institutions. Robust testing and documentation can significantly improve reproducibility in this respect, and guidelines such as those used by the Journal of Open Source Software (https://joss.readthedocs.io/en/latest/review_criteria.html) and the Journal of Open Research Software (https://openresearchsoftware.metajnl.com/about/editorialpolicies) can help support good testing practices.

### Publication

Add the links to data and code repositories to the manuscript. If possible, add the specific links to the data and code used to create the graphs in their caption to enable readers to “recalculate the figure from all its data, parameters, and programs”.^60^ Upload the paper to a preprint archive, such as arXiv (www.arxiv.org), bioRxiv (www.biorxiv.org), or medRxiv (www.medrxiv.org), and then submit it to a journal that supports open access. In the not too distant future, we may be able to write fully executable and interactive papers in which readers can rerun the described experiments.^13^ This vision is supported by existing pilot projects, where Jupyter Notebooks are submitted together with conference abstracts.^61^^,^^62^

### Licenses

Finally, add a license to your data, code, and publication to specify how you want them to be reused and modified. Without a license, you would retain all the rights and other researchers would not be allowed to use your material.^27^ If available, use the licenses recommended by your institution. Otherwise, the ORMIR guidelines are reported below.

For data and publication, use the Creative Commons (CC) license, whose features—attribution (BY), Non Commercial use (NC), Share-Alike (SA), and No Derivative (ND)—can be combined to create a customized license. Creative Commons also provides the CC0 license, which permits releasing material to the public domain without retaining any copyright (https://creativecommons.org/share-your-work/cclicenses).

For code, two main types of open-source licenses exist: restrictive—which extend openness to derived work (eg, GNU licenses)—and permissive, which do not require openness for derived work (eg, MIT, BSD, or Apache). For a complete list of licenses with their permissions and limitations, see https://choosealicense.com/appendix. Choose a license that is compatible with the licenses of the computational tools you used in your study and that you consider the most appropriate for the future of your project.^22^

## Just start!

Open and reproducible research is a way of working that supports our needs as researchers to assess scientific claims, build on previous reliable work, and contribute to faster advancement of our research field. In this perspective paper, we proposed concrete guidelines created by the ORMIR community on how to work in an open and reproducible manner. Adopting this methodological approach is a learning process that is most effective when learned by doing. If you have doubts about some guidelines or tools, contact the ORMIR community. Do not aspire to perfection from the very beginning. Just start! Your efforts will prove valuable because other researchers will be able to deeply understand and build on your research, and you will have contributed to a more transparent and faster advancement of MSK imaging research.

## Data Availability

No data were generated or analyzed in this paper.
